# Supplementary material for: Pre-activation of autophagy impacts response to olaparib in prostate cancer cells
Source: Commun Biol. 2022 Mar 22;5:251. doi: 10.1038/s42003-022-03210-5 (PMC8940895; doi:10.1038/s42003-022-03210-5)
Supplement: Supplementary file 2 — Supplementary Information [file 42003_2022_3210_MOESM2_ESM.pdf]

Pre-activation of autophagy impacts response to olaparib in prostate cancer cells

Maxime Cahuzac[1,2], Patricia Langlois[1,2], Benjamin Péant[1,2], Hubert Fleury[1,2], Anne-Marie Mes-Masson[1,2,3] and Fred Saad[1,2,3]

[1] Centre de recherche du Centre hospitalier de l'Université de Montréal (CRCHUM), Montreal, QC, Canada

[2] Institut du cancer de Montréal, Montreal, QC, Canada

[3] Department of Medecine, Université de Montréal, Montreal, QC, Canada

Corresponding author:

Anne-Marie Mes-Masson PhD, FCAHS, FRSC

Centre de recherche du CHUM, 900 rue Saint-Denis, Montreal QC H2X 0A9

anne-marie.mes-masson@umontreal.ca

514 890-8000, poste 25496

SUPPLEMENTARY INFORMATION

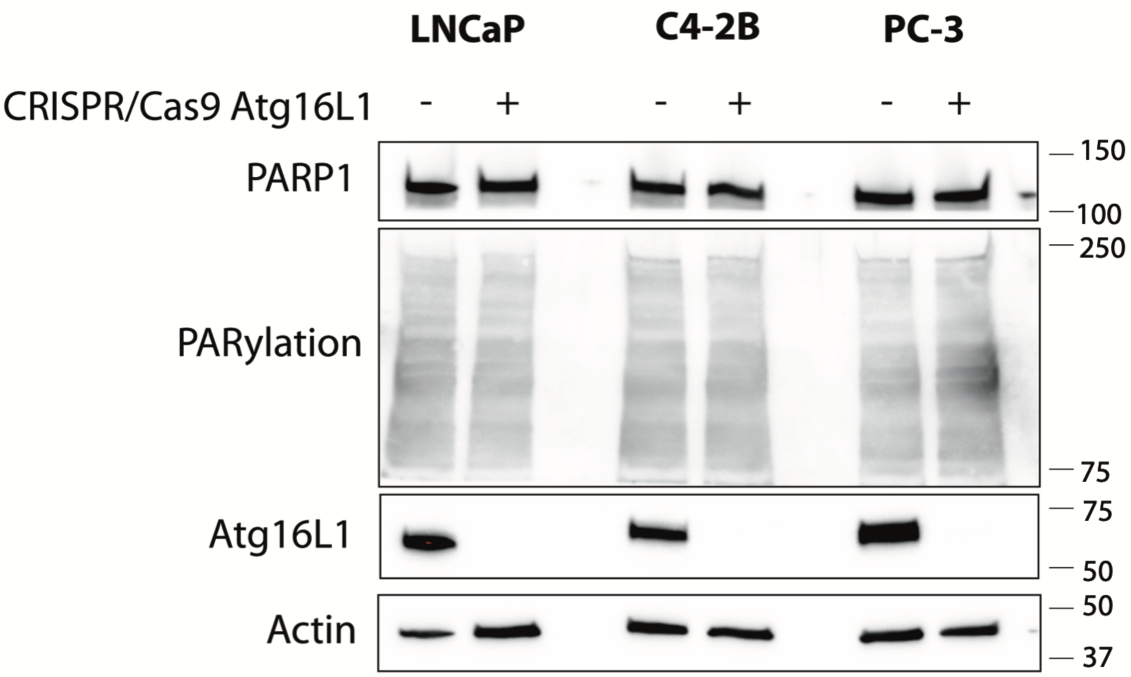

Supplementary Figure 1: Depletion of autophagy does not affect PARP1 expression and PARylation. Western blot analyses of PARP1 expression and PARylation between PC WT and KO *Atg16L1* cell lines.

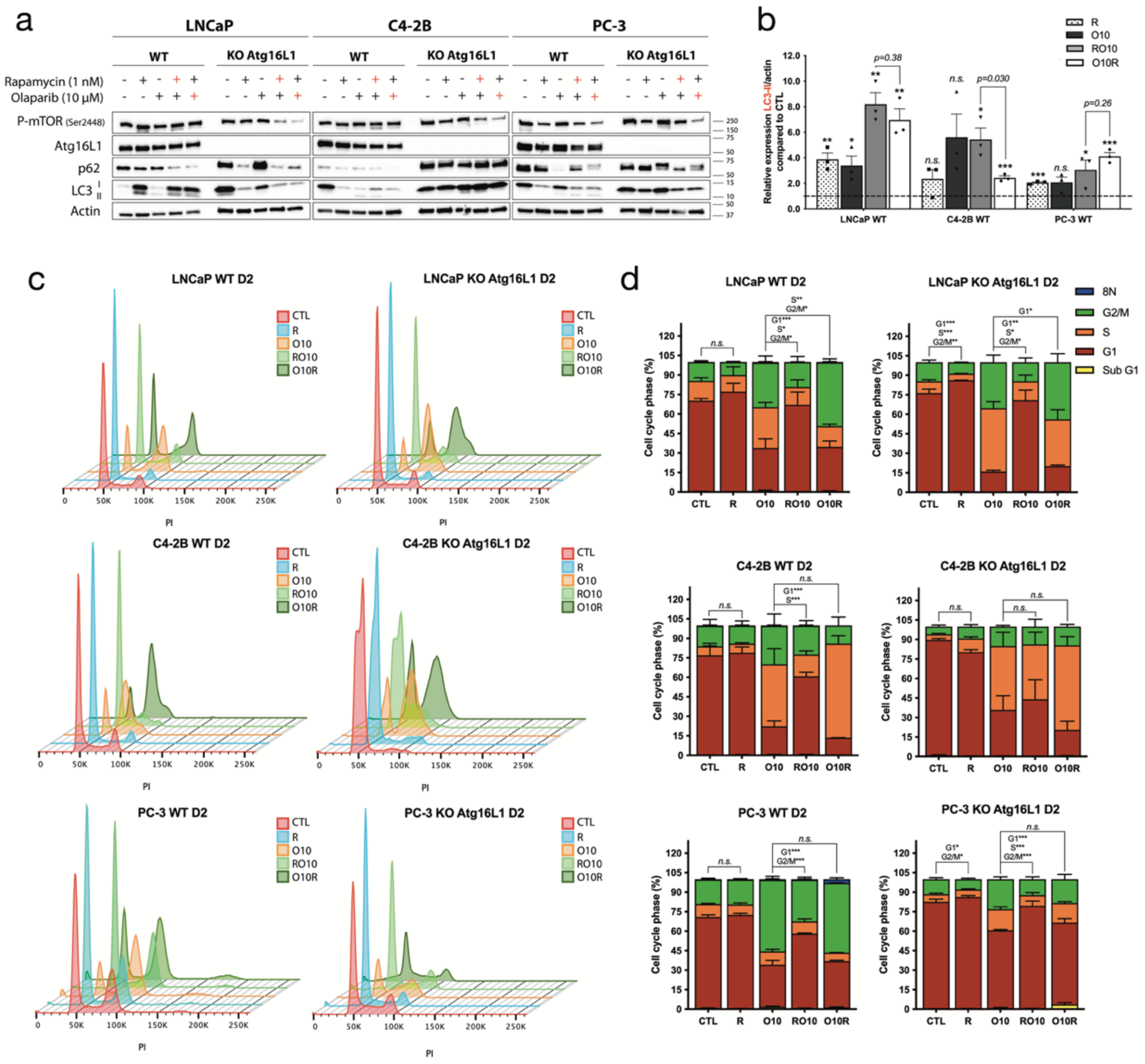

Supplementary Figure 2: Pre-activation of autophagy by rapamycin decreases effects of olaparib on cell cycle at day 2. (**a**). Western blot analyses of autophagy induction after olaparib and rapamycin treatments in WT and KO cell lines at day 6 performed at the same time as Fig. 3b. Rapamycin (+) denotes RO10. Olaparib (+) denotes O10R. (**b**). Relative expression of LC3-II normalized with actin and compared to control (CTL) from (a). (**c**). Representative flow analysis of DNA content (PI) for cell cycle measurement following 2 days of treatment of WT and KO PC cell lines (**d**). Quantification of (c). For all data, the mean ± SEM of three independent experiments is shown. Data were analyzed using the two-tail Student t-test. *p < 0.05, **p < 0.01, and ***p < 0.001.

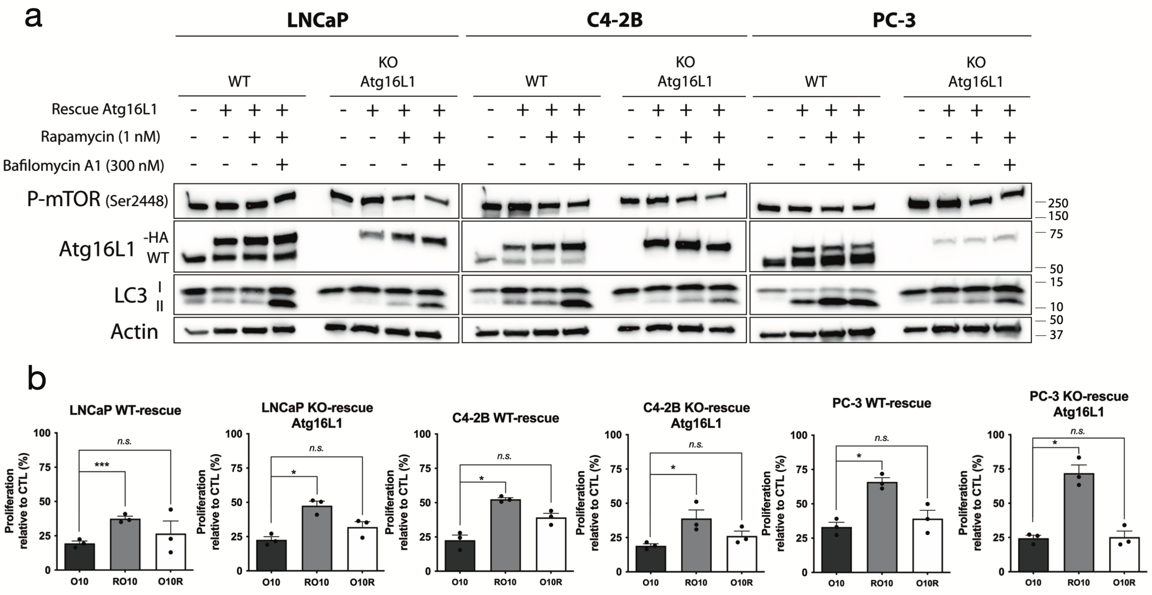

Supplementary Figure 3: Rescue of Atg16L1 expression restore autophagy-mediated resistance in PC KO *Atg16L1* cell lines. (**a**). Confirmation of restoration of autophagy after Atg16L1-HA rescue in PC WT and KO *Atg16L1* cell lines under control, autophagy-induced and autophagy-blocked conditions by western blot analysis. (**b**). Cell proliferation of WT-rescue *Atg16L1* and KO-rescue *Atg16L1* cell lines under O10, RO10 or O10R treatments at day 6. For all data, the mean ± SEM of three independent experiments is shown. Data were analyzed using the two-tail Student t test. n.s. = non-significant. *p < 0.05 and ***p < 0.001.

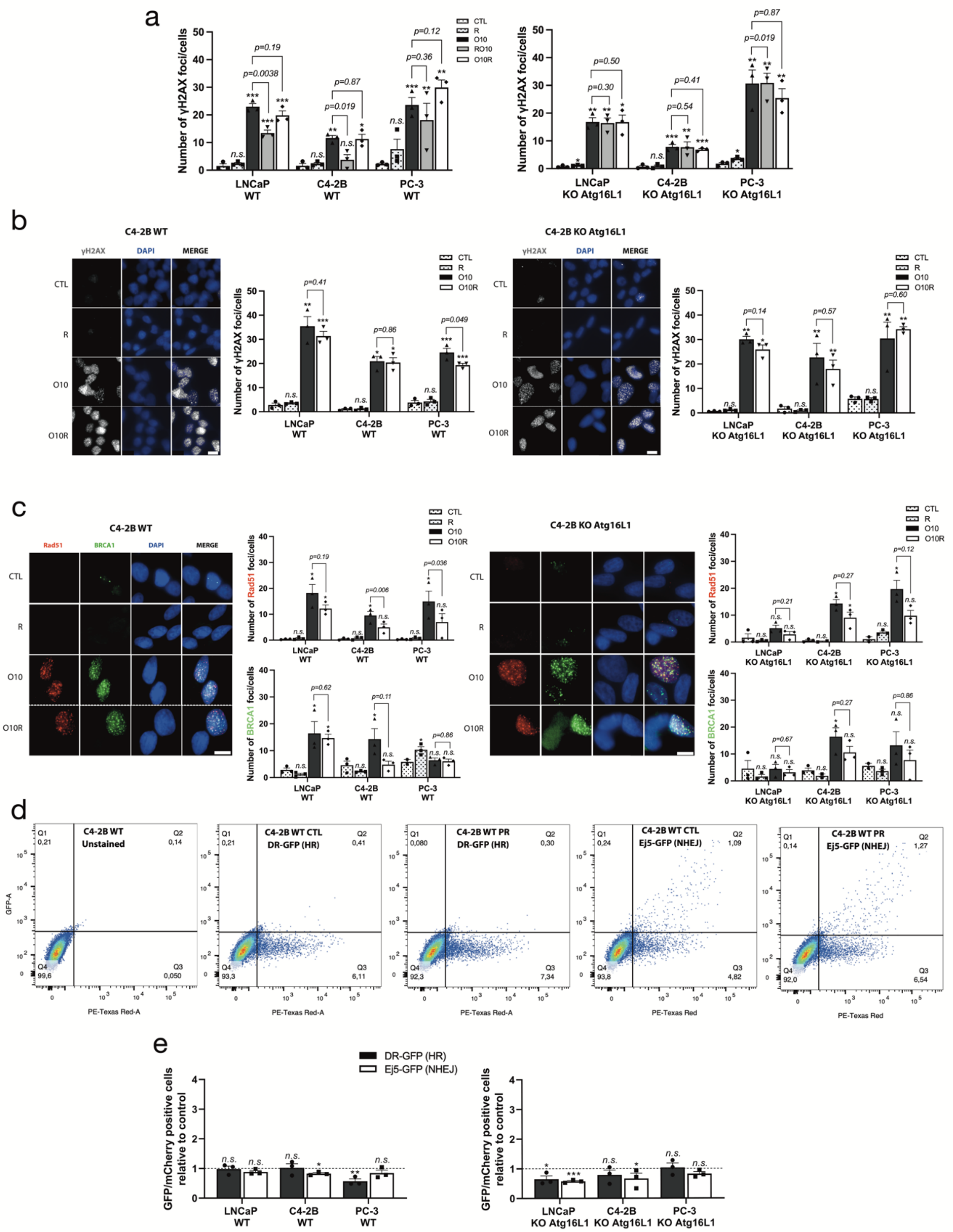

Supplementary Figure 4: Autophagy activation after olaparib treatment does not increase HR activity. (**a**). Quantification of γ-H2AX foci in WT and *Atg16L1* KO cell lines following treatment with rapamycin alone (R), O10 or RO10 after 6 days. (**b**). Representative images (C4-2B WT and KO *Atg16L1*) and quantification of number of

γ-H2AX foci per nucleus cell lines following treatment of rapamycin alone (R), O10 or O10R treatments after 2 days. (**c**). Representative images (C4-2B WT and KO *Atg16L1*) and quantification of Rad51 and BRCA1 foci following same conditions as (b). (**d**). Representative gating strategy to quantification of HR (DR-GFP) and NHEJ (Ej5-GFP) in unstained, control and when autophagy was activated by rapamycin 24 hours after plasmids co-transfection (TR) in C4-2B WT. (**e**). Quantification of (d). All experiments were performed at the same time as Fig. 3. For all data, the mean ± SEM of three independent experiments is shown. Data were analyzed using the two-tail Student t test. n.s. = non-significant. *$p < 0.05$, **$p < 0.01$, and ***$p < 0.001$. Scale bar 10 µm.

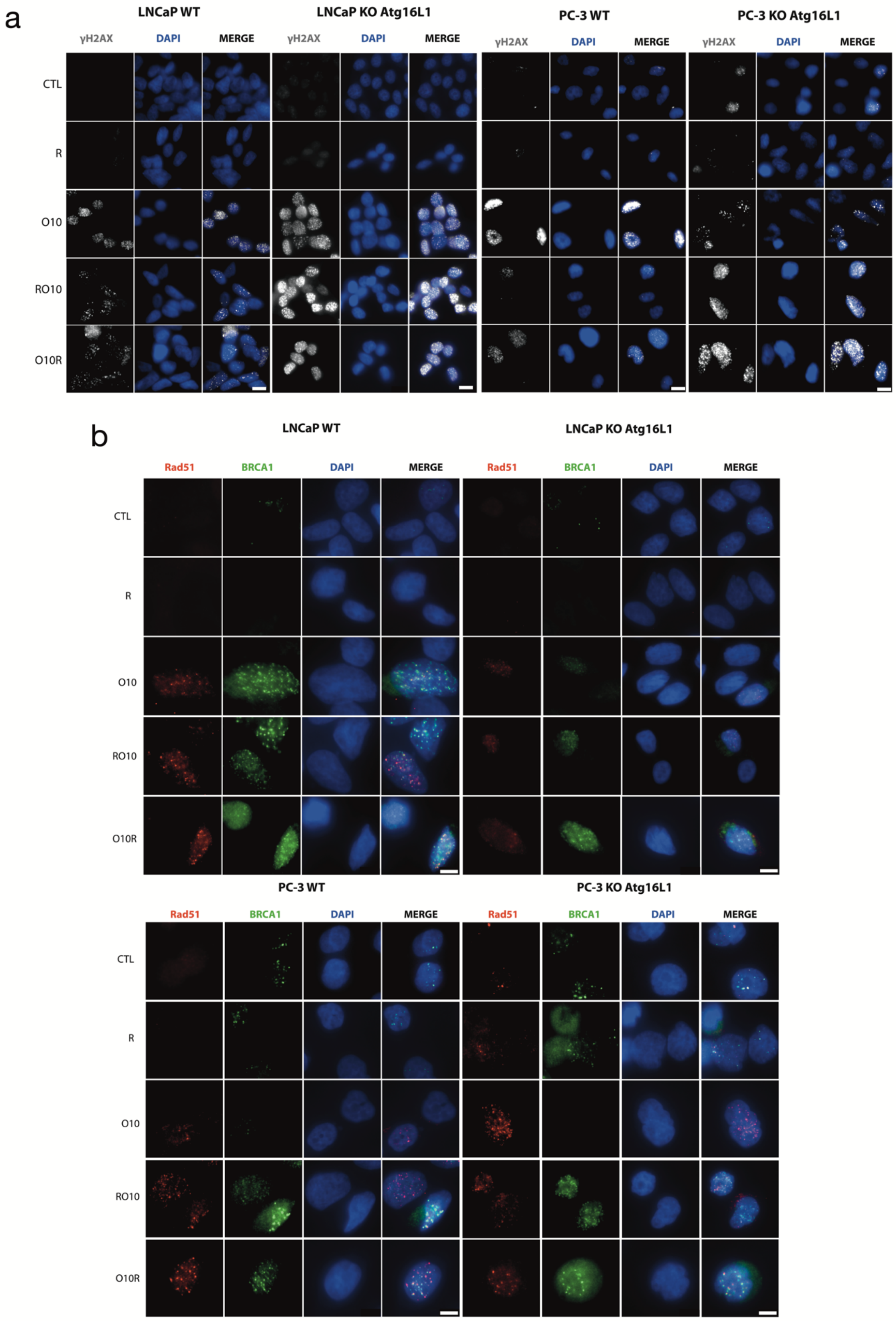
a
LNCaP WT
LNCaP KO Atg16L1
PC-3 WT
PC-3 KO Atg16L1
γH2AX
DAPI
MERGE
CTL
R
O10
RO10
O10R
b
LNCaP WT
LNCaP KO Atg16L1
Rad51
BRCA1
DAPI
MERGE
CTL
R
O10
RO10
O10R
PC-3 WT
PC-3 KO Atg16L1

Supplementary Figure 5: Autophagy pre-activation leads to an increase of Rad51/BRCA1 recruitment and H2AX resolution. (**a**). Representative images of number of γ-H2AX foci per nucleus in LNCaP and PC-3 WT and KO cells following Fig. 3 conditions. (**b**). Representative images of number of Rad51 and BRCA1 foci per nucleus in the same PC cell lines and following Fig. 3 conditions. Scale bar 10 μm.

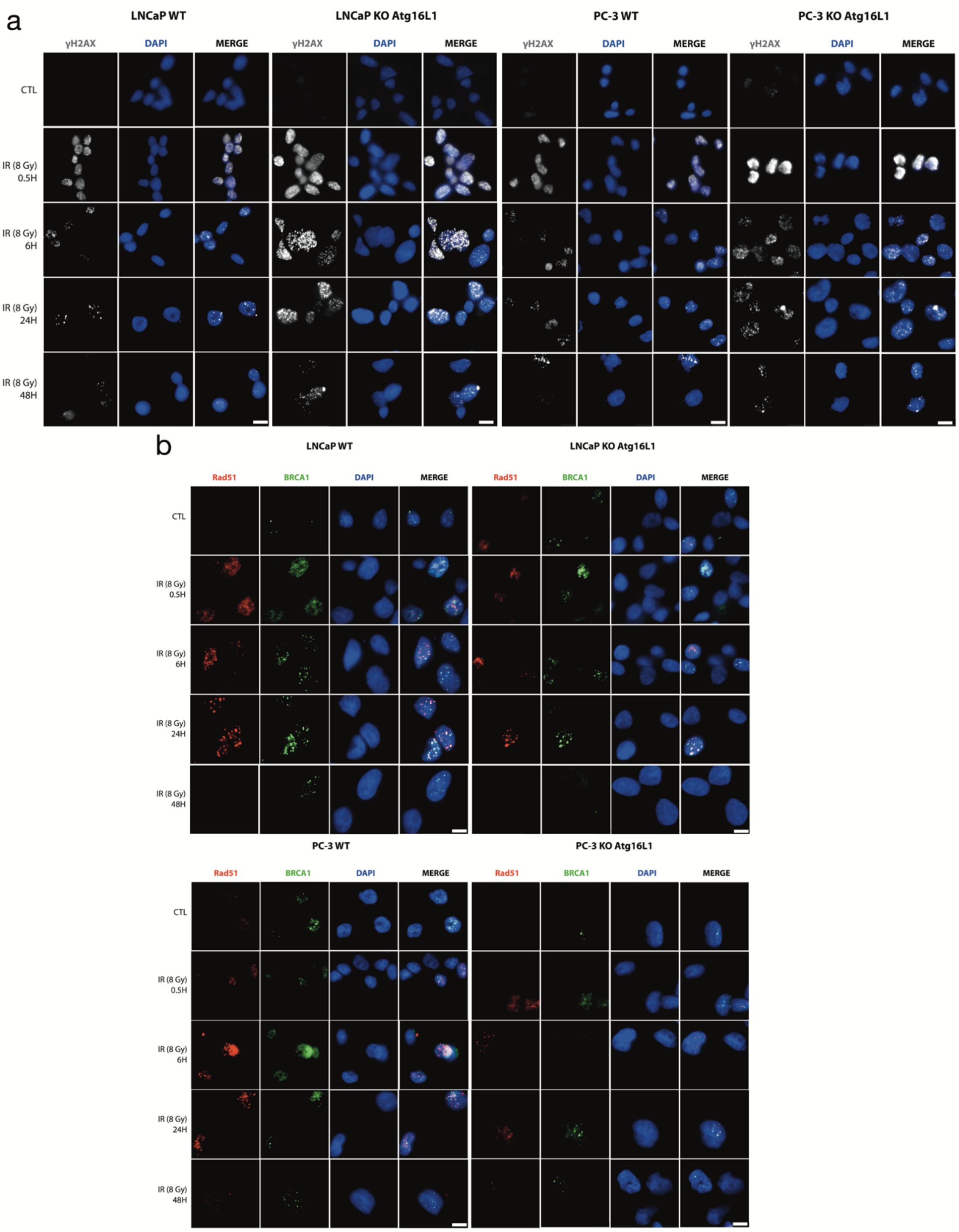

Supplementary Figure 6: Autophagy-depleted PC cells have a lower recruitment of BRCA1/Rad51 to repair DNA breaks. (**a**). Representative images of number of γ-H2AX foci per nucleus in WT and KO cells of LNCaP and PC-3 following Fig. 4

conditions. (**b**). Representative images of number of Rad51 and BRCA1 foci per nucleus in WT and KO cells of LNCaP and PC-3 after 8 Gy irradiation. Scale bar 10 μm.

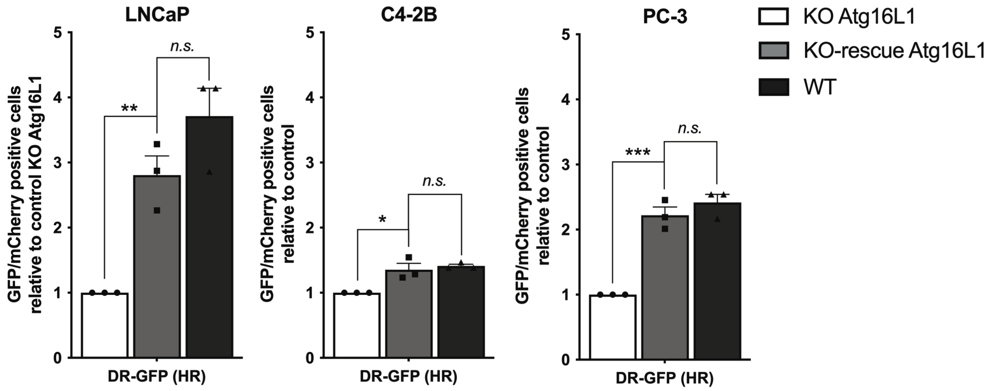

Supplementary Figure 7: Rescue of Atg16L1 restore DNA repair HR efficiency. Quantification of HR (DR-GFP) activity in PC WT and KO-rescue *Atg16L1* cell lines compared to PC KO *Atg16L1*. The ratio of GFP-positive cells versus mCherry-positive cells was determined by flow cytometry. For all data, the mean ± SEM of three independent experiments is shown. Data were analyzed using the two-tail Student t test. n.s. = non-significant. *p < 0.05, **p < 0.01, and ***p < 0.001.

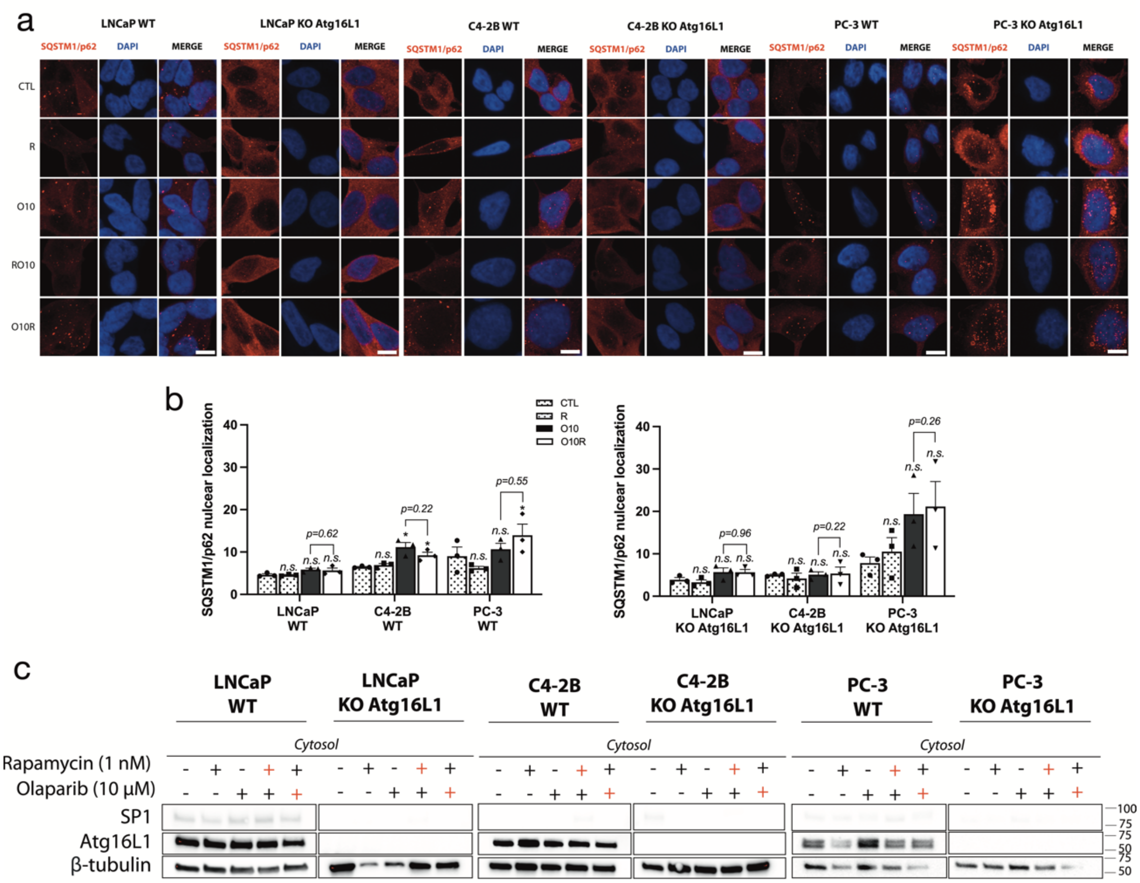

Supplementary Figure 8: SQSTM1/p62 nuclear localization regulates autophagy-mediated resistance to olaparib. (**a**). Representative images of number of SQSTM1/p62 puncta in WT and KO *Atg16L1* cell lines following rapamycin alone (R), O10 or O10R treatments after 2 days. (**b**). Quantification of nuclear SQSTM1/p62 in WT and KO cell lines in the same conditions as (a). (**c**). Western blot of cytoplasmic fraction after 2 days of RO10 and O10R treatment in PC WT and KO *Atg16L1* cell lines. Each cytoplasmic fraction was loaded in the same gel as nuclear fraction in Fig. 5. ß-tubulin was used as quality control as marker of cytoplasmic fraction, SP1 as a marker of nuclear fraction and Atg16L1 as a confirmation of PC KO *Atg16L1* cell lines. For all data, the mean ± SEM of three independent experiments is shown. Data were analyzed using the two-tail Student t test. n.s. = non-significant. $^*p < 0.05$.

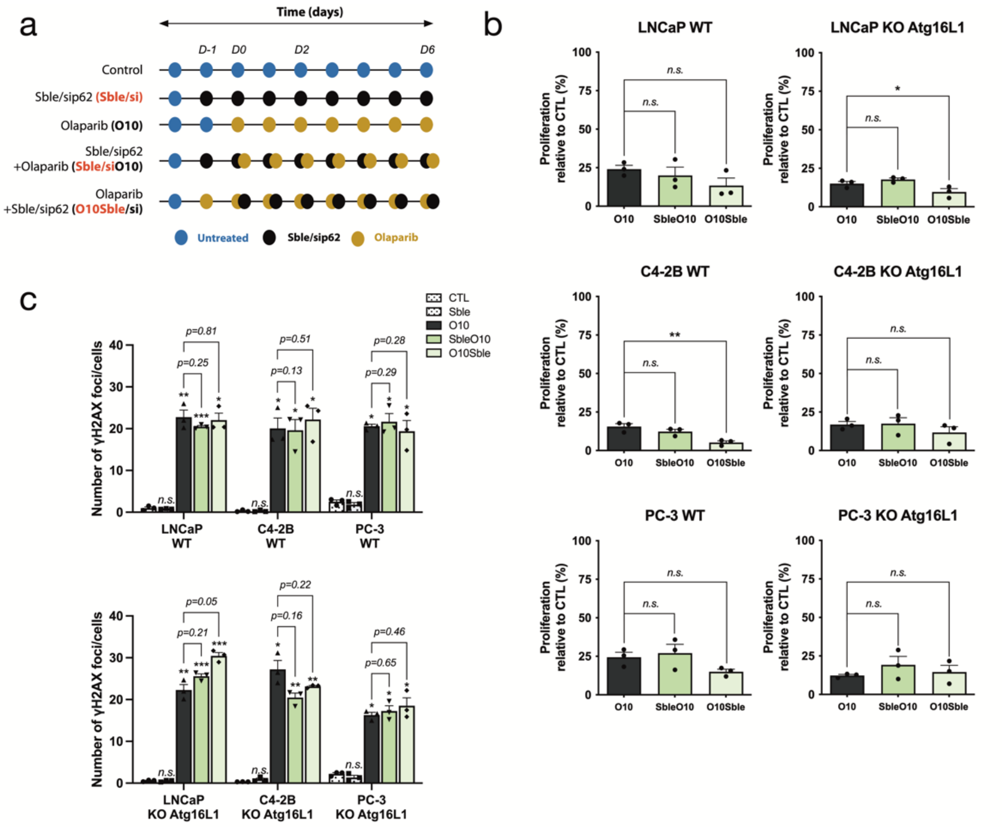

Supplementary Figure 9: Scramble transfection does not affect cell proliferation or γ-H2AX foci resolution. (**a**). Transfection timeline of cell lines. Cells were treated with 10 µM olaparib alone (denoted as O10) or transfected with siRNA against SQSTM1/p62 (si) or with scramble (Sble 24 hours before (Sble/siO10) or 24 hours after (O10Sble/si) the start of olaparib treatment. Experiments were conducted for 6 days. (**b**). Cell proliferation of PC WT and KO *Atg16L1* cell lines in SbleO10 and O10Sble conditions at day 6, performed at the same time as Fig.6c. (**c**). Quantification of the number of γ-H2AX foci per nucleus in PC WT-rescue and KO-rescue cell lines following transfection with scramble (Sble) or 24 hours before olaparib treatment (SbleO10) or after (O10Sble) at day 2. All Sble conditions were performed at the same time as all siRNA conditions. For all data, the mean ± SEM of three independent experiments is shown. Data were analyzed using the two-tail Student t test. n.s. = non-significant. *p < 0.05, **p < 0.01, and ***p < 0.001.

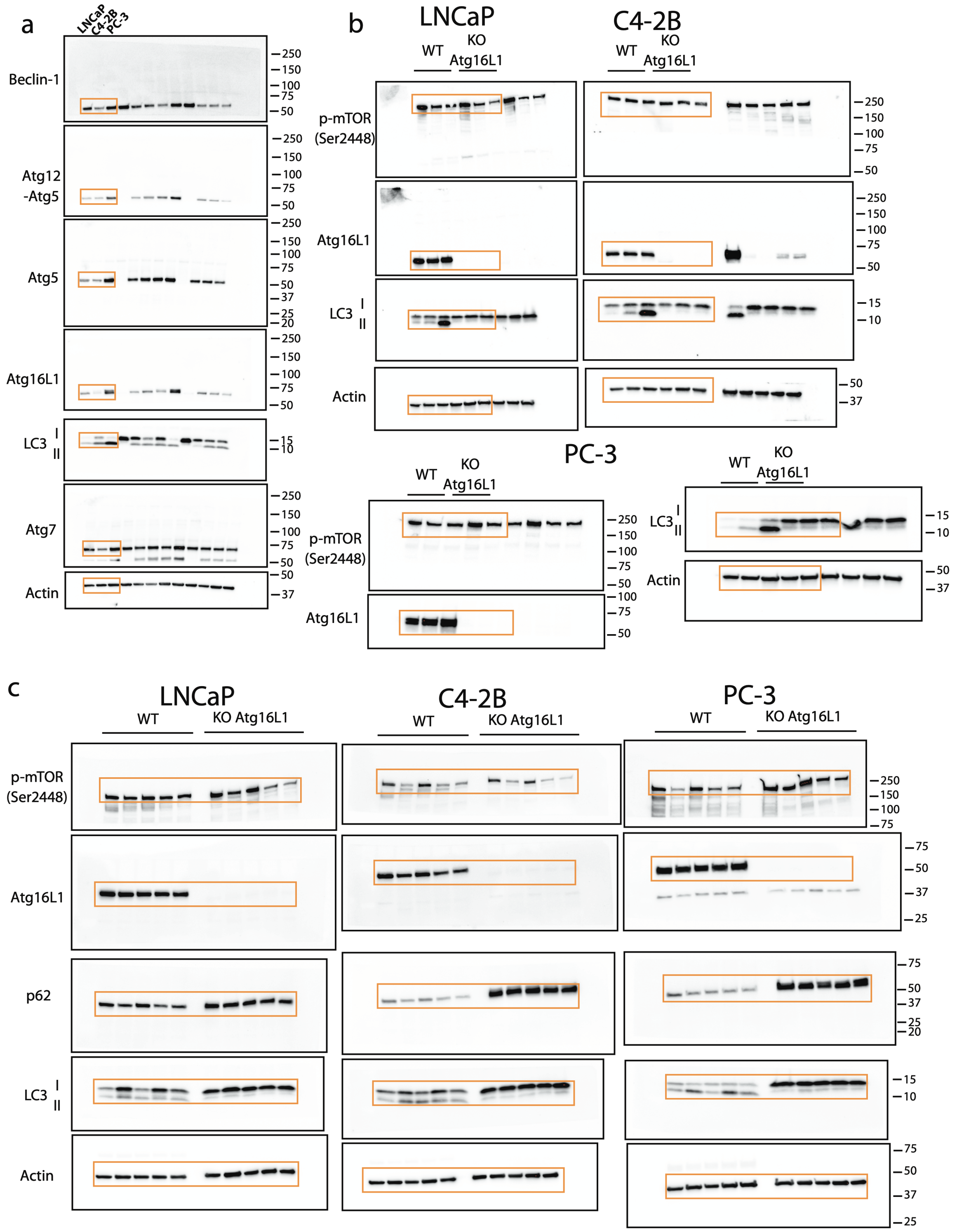
a
LNCaP
C4-2B
PC-3
Beclin-1
Atg12
-Atg5
Atg5
Atg16L1
LC3 I
II
Atg7
Actin
b
LNCaP
C4-2B
WT
KO
Atg16L1
p-mTOR
(Ser2448)
Atg16L1
LC3 I
II
Actin
PC-3
c
LNCaP
C4-2B
PC-3
WT
KO Atg16L1
p-mTOR
(Ser2448)
Atg16L1
p62
LC3 I
II
Actin

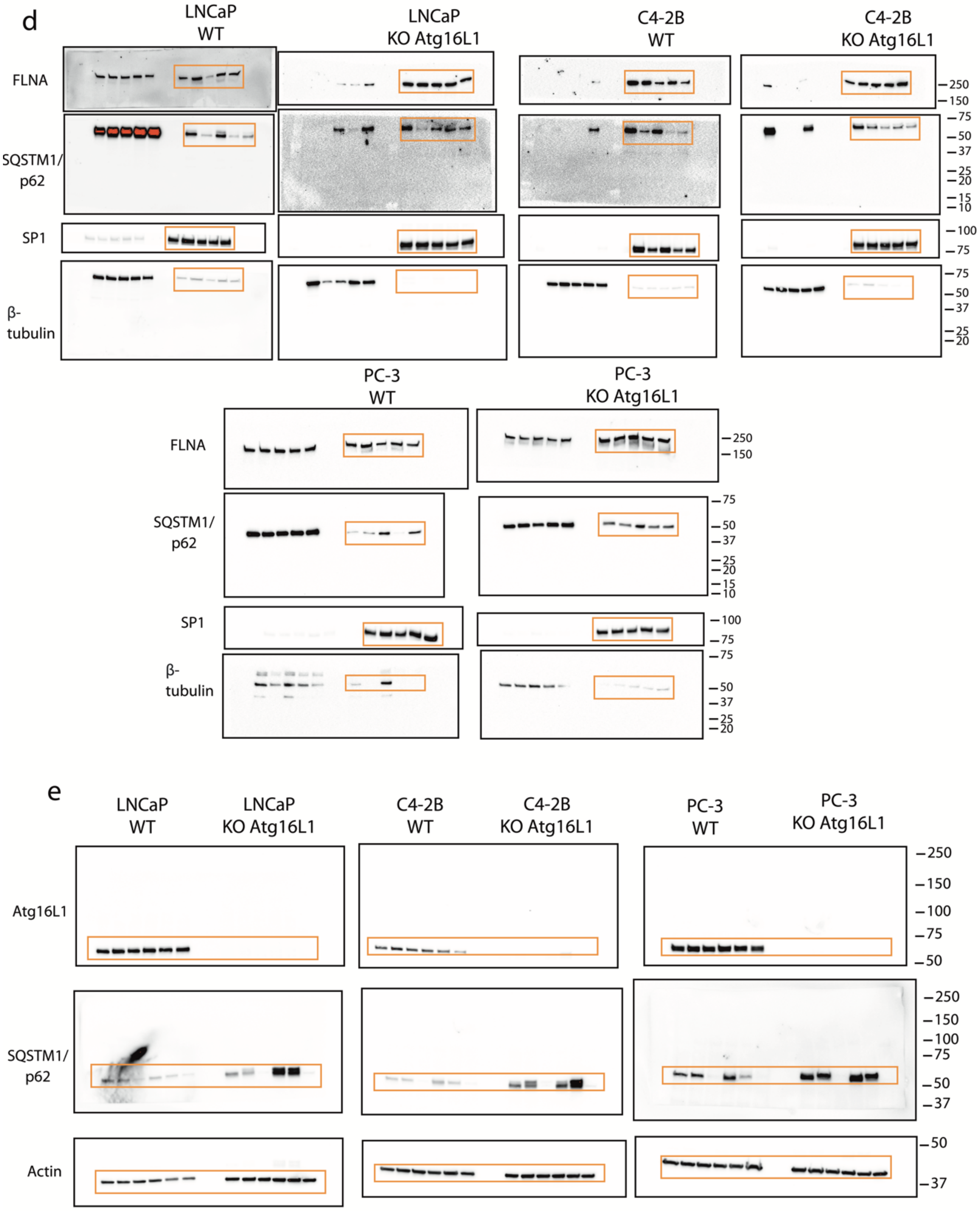

Supplementary Figure 10: Uncropped blots. **(a)** Blots from Figure 1b. **(b)** Blots from Figure 1f. **(c)** Blots from Figure 2b. **(d)** Blots from Figure 5c. **(e)** Blots from Figure 6a.
