# Supplementary material for: Pre-activation of autophagy impacts response to olaparib in prostate cancer cells
Source: Commun Biol. 2022 Mar 22;5:251. doi: 10.1038/s42003-022-03210-5 (PMC8940895; doi:10.1038/s42003-022-03210-5)
Supplement: Supplementary file 3 — Description of Additional Supplementary Files [file 42003_2022_3210_MOESM3_ESM.pdf]

Description of Additional Supplementary Files

File name: Supplementary Data 1
Description: Source data presented in the figures.
