# Supplementary material for: Pre-activation of autophagy impacts response to olaparib in prostate cancer cells
Source: Commun Biol. 2022 Mar 22;5:251. doi: 10.1038/s42003-022-03210-5 (PMC8940895; doi:10.1038/s42003-022-03210-5)
Supplement: Supplementary file 5 — Reporting Summary [file 42003_2022_3210_MOESM5_ESM.pdf]

nature portfolio

Corresponding author(s): Anne-Marie Mes-Masson
Last updated by author(s): Feb 16, 2022

Reporting Summary

Nature Portfolio wishes to improve the reproducibility of the work that we publish. This form provides structure for consistency and transparency in reporting. For further information on Nature Portfolio policies, see our Editorial Policies and the Editorial Policy Checklist.

Statistics

For all statistical analyses, confirm that the following items are present in the figure legend, table legend, main text, or Methods section.

n/a	Confirmed	
☐	☒	The exact sample size (*n*) for each experimental group/condition, given as a discrete number and unit of measurement
☐	☒	A statement on whether measurements were taken from distinct samples or whether the same sample was measured repeatedly
☐	☒	The statistical test(s) used AND whether they are one- or two-sided<br>*Only common tests should be described solely by name; describe more complex techniques in the Methods section.*
☒	☐	A description of all covariates tested
☐	☒	A description of any assumptions or corrections, such as tests of normality and adjustment for multiple comparisons
☐	☒	A full description of the statistical parameters including central tendency (e.g. means) or other basic estimates (e.g. regression coefficient) AND variation (e.g. standard deviation) or associated estimates of uncertainty (e.g. confidence intervals)
☒	☐	For null hypothesis testing, the test statistic (e.g. *F*, *t*, *r*) with confidence intervals, effect sizes, degrees of freedom and *P* value noted<br>*Give P values as exact values whenever suitable.*
☒	☐	For Bayesian analysis, information on the choice of priors and Markov chain Monte Carlo settings
☒	☐	For hierarchical and complex designs, identification of the appropriate level for tests and full reporting of outcomes
☒	☐	Estimates of effect sizes (e.g. Cohen's *d*, Pearson's *r*), indicating how they were calculated

Our web collection on statistics for biologists contains articles on many of the points above.

Software and code

Policy information about availability of computer code

Data collection	AxioVision, Rel. 4.9.1 (Carl Zeiss®), IncuCyteTM Live-Cell Imaging System (IncuCyte HD), Leica TCS-SP5 and Las-AF software, Fortessa flow cytometer (BD Biosciences).
Data analysis	Graphad 9, Flow Jo, Axiovision 4.9, ImageJ (find maxima, quantification western blot and analyze particules process and a macro adapted from Daniel J. Shiwarski as described in the manuscript), IncuCyteTM Live-Cell software, Microsoft Excel 2019, iDraw

For manuscripts utilizing custom algorithms or software that are central to the research but not yet described in published literature, software must be made available to editors and reviewers. We strongly encourage code deposition in a community repository (e.g. GitHub). See the Nature Portfolio guidelines for submitting code & software for further information.

Data

Policy information about availability of data

All manuscripts must include a data availability statement. This statement should provide the following information, where applicable:

- Accession codes, unique identifiers, or web links for publicly available datasets
- A description of any restrictions on data availability
- For clinical datasets or third party data, please ensure that the statement adheres to our policy

The authors declare that the data supporting the findings of this study are available within the paper and its supplementary information files. If needed, additional information is available from the corresponding author upon reasonable request.

Field-specific reporting

Please select the one below that is the best fit for your research. If you are not sure, read the appropriate sections before making your selection.

☒ Life sciences ☐ Behavioural & social sciences ☐ Ecological, evolutionary & environmental sciences

For a reference copy of the document with all sections, see nature.com/documents/nr-reporting-summary-flat.pdf

Life sciences study design

All studies must disclose on these points even when the disclosure is negative.

Sample size	The chosen sample size is based on numbers used for previous publiations, which is most optimal to generate statisticaly significant results.
Data exclusions	No data were excluded.
Replication	All attempts at replication were successful.
Randomization	Sample/Cells were randomized to be examined.
Blinding	Blinding was not relevant because all samples/cells were analyzed in the same way

Reporting for specific materials, systems and methods

We require information from authors about some types of materials, experimental systems and methods used in many studies. Here, indicate whether each material, system or method listed is relevant to your study. If you are not sure if a list item applies to your research, read the appropriate section before selecting a response.

Materials & experimental systems

n/a	Involved in the study	
☐	☒	Antibodies
☐	☒	Eukaryotic cell lines
☒	☐	Palaeontology and archaeology
☒	☐	Animals and other organisms
☒	☐	Human research participants
☒	☐	Clinical data
☒	☐	Dual use research of concern

Methods

n/a	Involved in the study	
☒	☐	ChIP-seq
☐	☒	Flow cytometry
☒	☐	MRI-based neuroimaging

Antibodies

Antibodies used	The following antibodies were used for the study: Beta-Actin (AC14) (abcam, AB6276); Atg16L1 (D6D5) (Cell Signaling, 8089T); anti-phospho-Histone H2A.X (Ser139) (Sigma-Aldrich, JBW301); LC3 A/B (D3U4C) (Cell Signaling, 12741S); phospho-mTOR (Ser2448) (D9C2) (Cell Signaling, 5536T); SQSTM1/p62 (Cell Signaling, 5114T) and (abcam, ab56416); Rad51 (114B4) (abcam, ab213); and BRCA1 (Sigma Millipore, 07-434); Atg5 (D5F5U) (Cell Signaling, 12994S); Atg12 (D88H11) (Cell Signaling, 4180S); Beclin-1 (D40C5) (Cell Signaling, 3495S); Atg7 (D12B11) (Cell Signaling, 8558S); β-Tubulin (D2N5G) (Cell Signaling, 15115S); Filamin A (Cell Signaling, 4762S), SP1 (Sigma, PLA0307), PARP1 (proteintech, 66250), PAR/pADR (R&D systems, 4335-MC-100), Goat anti-mouse (Millipore, AP124P), Goat anti-rabbit (Millipore, AP156P), Rabbit anti-goat (Milliporte, AP106P), anti-mouse Cy5 (Life Technologies Inc., A10524) and anti-rabbit 488 (ThermoFisher Scientific, A-11008).
Validation	Antibody validation was deferred to the manufacturers and was supported by multiple publications.

Eukaryotic cell lines

Policy information about cell lines

Cell line source(s)	Human PC cell lines, LNCaP and PC-3, were purchased from the American Type Culture Collection (ATCC CRL-174, ATCC CRL-250, respectively). C4-2B cells were gifted by Dr. Gleave. PC CRISPR KO Atg16L1 cell lines were developed from the parental ones.
Authentication	PC cell lines were authenticated by Short Tandem Repeat (STR) profiling
Mycoplasma contamination	All cell lines were tested negative for mycoplasma.

Commonly misidentified lines
(See ICLAC register)

No commonly misidentified cell lines were used in this study.

Flow Cytometry

Plots

Confirm that:

☒ The axis labels state the marker and fluorochrome used (e.g. CD4-FITC).

☒ The axis scales are clearly visible. Include numbers along axes only for bottom left plot of group (a 'group' is an analysis of identical markers).

☒ All plots are contour plots with outliers or pseudocolor plots.

☒ A numerical value for number of cells or percentage (with statistics) is provided.

Methodology

Sample preparation	Cell lines
Instrument	BD LSRFortessa
Software	Flow-Jo
Cell population abundance	A maximum of 30,000 events was counted for cell cycle, 15,000 for HR/NHEJ analyzes and 10,000 for siRNA experiement
Gating strategy	We removed element under 50 FSC and under 50 SSC which are considered as debris. Under FSC-H/FSC-W we gate the low population to eliminated doublet.

☒ Tick this box to confirm that a figure exemplifying the gating strategy is provided in the Supplementary Information.
